# PERCEIVED NEIGHBORHOOD RESOURCES AND MEDIATING ROLE OF SOCIAL COHESION ON FUNCTIONAL HEALTH AMONG VERY OLD ADULTS

**DOI:** 10.1093/geroni/igae098.0283

**Published:** 2024-12-31

**Authors:** Jaroslava Zimmermann, Ibrahim Demirer

**Affiliations:** University of Cologne, Cologne, Nordrhein-Westfalen, Germany; University of Cologne, Faculty of Medicine and University Hospital Cologne, Institute of Medical Sociology, Health Services Research, and Rehabilitation Science (IMVR), Cologne, Nordrhein-Westfalen, Germany

## Abstract

Functional health is not only a key indicator of physical health in old age, but it also reflects the individual ability to self-care and participate independently in important areas of life. The evidence shows that older adults living in socioeconomically deprived neighborhoods have higher likelihood to experience functional limitations. Nevertheless, little is known about the underlying mechanisms explaining this effect. Following the social disorganization theory, the aim of this contribution was to test the hypothesis that the link between neighborhood socioeconomic resources and functional health is mediated by social cohesion. Data from the German representative study “Old age in Germany” were used. The study sample included 10,578 persons aged ≥80 years. Functional health was measured by instrumental activities of daily living (IADL). Neighborhood socioeconomic resources were operationalized by perceived walkability of neighborhood and building condition. Social cohesion referred to trusting people in neighborhood. Considering the complex sample design, path analysis based on structure equation modeling was conducted. Adjusting for individual and regional characteristics, the results showed that low walkability and poor building condition (independently of each other as well as in a combined form) were associated with decreased ability in IADL and lower social cohesion, while higher social cohesion increased IADL. The findings suggest that unfavorable socioeconomic conditions in neighborhoods negatively affect social cohesion and functional health. Moreover, social cohesion in neighborhoods have protective effect on functional health. Both, socioeconomic and social conditions should be targeted when planning interventions to promote or maintain functional health of the very old.

